# Development of a core outcome set for informed consent for therapy: An international key stakeholder consensus study

**DOI:** 10.1186/s12910-022-00820-w

**Published:** 2022-08-09

**Authors:** Liam J. Convie, Joshua M. Clements, Scott McCain, Jeffrey Campbell, Stephen J. Kirk, Mike Clarke

**Affiliations:** 1 Department of General Surgery, Ulster Hospital, Upper Newtownards Road, Dundonald, Belfast, BT16 1RH UK; 2 Centre for Public Health, Institute of Clinical Sciences, Royal Victoria Hospital, Belfast, BT12 6BA UK

**Keywords:** Informed consent, Core outcome set, Surgery

## Abstract

**Background:**

300 million operations and procedures are performed annually across the world, all of which require a patient’s informed consent. No standardised measure of the consent process exists in current clinical practice. We aimed to define a core outcome set for informed consent for therapy.

**Methods:**

The core outcome set was developed in accordance with a predefined research protocol and the Core OutcoMes in Effectiveness Trials (COMET) methodology comprising systematic review, qualitative semi structured interviews, a modified Delphi process and consensus webinars to ratify outcomes for inclusion in the final core outcome set. (Registration—https://www.comet-initiative.org/Studies/Details/1024). Participants from all key stakeholder groups took part in the process, including patients and the public, healthcare practitioners and consent researchers.

**Results:**

36 outcome domains were synthesised through systematic review and organised into a consent taxonomy. 41 semi-structured interviews were performed with all consent stakeholders groups. 164 participants from all stakeholder groups across 8 countries completed Delphi Round 1 and 125 completed Round 2. 11 outcomes met the ‘consensus in’ criteria. 6 met ‘consensus in’ all stakeholder groups and were included directly in the final core outcome set. 5 remaining outcomes meeting ‘consensus in’ were ratified over two consensus webinars. 9 core outcomes were included in the final core outcome set: Satisfaction with the quality and amount of information, Patient feeling that there was a choice, Patient feeling that the decision to consent was their own, Confidence in the decision made, Satisfaction with communication, Trust in the clinician, Patient satisfaction with the consent process, Patient rated adequacy of time and opportunity to ask questions.

**Conclusion:**

This international mixed-methods qualitative study is the first of its kind to define a core outcome set for informed consent for intervention. It defines what outcomes are of importance to key stakeholders in the consent process and is a forward step towards standardising future consent research.

**Supplementary Information:**

The online version contains supplementary material available at 10.1186/s12910-022-00820-w.

## Background

Over 300 million operations and procedures take place around the world annually. All these procedures require the patient to give informed consent [1]. Consent, alongside shared decision making are cornerstones of Good Medical Practice as outlined by the General Medical Council (GMC) [2]. Consent is an integral part of medical and public health ethics and international law. Failings in the informed consent process can lead to dissolution of the clinician-patient relationship, complaints and occasionally litigation [3]. In the United Kingdom alone, National Health Service (NHS) England compensated patients £134.5 million between 2013 and 2018 in cases relating directly to deficiencies in the informed consent process [4].

Numerous studies exist evaluating the effects of various techniques (e.g. audio-visual/multimedia assisted consent) designed to improve informed consent. However, systematic review of this evidence has highlighted the heterogeneous nature of data in terms of study design and the choice of outcome measures which ultimately limits the generation of consensus on which interventions are most effective. Only one study, at high risk of bias, has attempted to measure informed consent as a unified concept [5]. It has been highlighted that trialists should recognise the complexity of the informed consent process by considering the overall patterns of outcomes and not simply use a measure of knowledge that has often been the case previously. Therefore, there has been a call for greater consensus on appropriate, validated and reliable tools for assessing the effects of interventions for the consent process to facilitate comparison between studies and to enable the meaningful synthesis of results [6–9]. In addition, it is unclear as to whether the outcomes that have been measured in previous consent research are the things that are of importance to key stakeholders during the consent process.

Core outcome sets (COS) aim to define a minimum set of outcomes that should be considered essential in the evaluation and reporting of studies of a particular intervention or condition [10]. There are well-defined guidelines with a growing evidence base to support the use of COS and the methodology employed to develop them [10–15]. Increasingly, researchers are inviting different stakeholder groups to identify the important outcomes for future evaluations of interventions in a variety of health areas such as cancer, rheumatology and otorhinolaryngology [16–18]. These activities have demonstrated that each stakeholder group may rate the importance of outcomes differently, reflecting their own priorities [15, 19]. Additionally, these priorities may not always align with the priorities of researchers who have traditionally been in control of the outcomes being investigated.

Knowledge and with that patient understanding have been the predominantly measured primary outcomes in consent research however, there are a range of other issues that may matter to stakeholders in the process.

The development of a Core Outcome Set (COS) may help researchers select and measure the most relevant outcomes that are most important to stakeholders involved in the process [20]. The primary benefit of using a COS allows the most important outcomes to be consistently measured and reported, thus allowing; comparisons between studies, the synthesis of data in meta-analyses and a reduction in reporting bias [10].

The aim of this study was to define a COS to evaluate interventions to improve consent for surgery and other invasive procedures, in adult patients (over 18 years) with adequate mental capacity to make their own consent decisions.

## Methods

This study was developed in accordance with the guidance published in the Core Outcome Measures in Effectiveness Trials (COMET) Handbook and the Core Outcome Set-Standards for Development (COS-STAD) statement [10, 21]. The reporting of the study methods and findings has been undertaken in accordance with the Core Outcome Set-Standards for Reporting (COS-STAR) [13]. The protocol for the development of this COS was registered prospectively on the COMET database and published in full before work on the consensus building components of the project were undertaken [22, 23]. Prospective ethical approval for the study was obtained from the Office of Research Ethics Northern Ireland (RECA 17/NI/0234) and the Research and Development Office of the South Eastern Health and Social Care Trust (SET.17.36_SEHSCT). All methods were carried out in accordance with the ethical principles of the Declaration of Helsinki.

## Study advisory group (SAG) and patient and public involvement (PPI)

The study advisory group was formed from members of the authorship list (LMC, SMcC, SJK, MC, WJC) as well as an experienced patient participant who has worked with the Research & Development department of the South Eastern Health and Social Care Trust on a wide range of clinical trials and other clinical research for several years.

The GRIPP2-Short Form Checklist (Table 1) outlines the Patient Involvement in Research in this study [24]; Our methods are reflective of some of the learning points from previous studies defining and evaluating novel procedures for involving patients and the public in COS research [25]. We defined “Public involvement”, “Public participation” and “Public engagement” according to those definitions from the INVOLVE advisory group members document [26].

**Table 1:** The guidance for reporting involvement of patients and the public (GRIPP2-short form) checklist

Section and topic	Item	Reported on page no
Aims	This study sought to develop international consensus on a core outcome set for informed consent for therapy for adults over 18 years with capacity to consent for themselves	3–4
Methods	An experienced patient partner was recruited to the research team from the SEHSCT Research & development department. They served to ensure patient involvement throughout the COS development process. They were involved in refining the research questions and helped draft the PPI strategy which was built into the study ethical approval. This facilitated the SAG to budget for specific elements of COS development and make decisions regarding PPI involvement at each stage	5–13
Study results	PPI contributed to the study in numerous ways, including; The patient partner provided feedback on the initial findings from our systematic review [27] of patient experiences which helped to define a long list of outcomes to be brought forward to the semi-structured interview stage of the COS. They provided lay feedback on the generation and wording of the Delphi Survey questions. Additionally, expert patient opinion was sought from the COMET PoPPIE group for feedback on patient engagement summary videos prior to release. Outcomes from the Delphi that were brought forward to consensus were discussed with the Royal college of surgeons of England Patient liaison group of patient experts	14–22
Discussion and conclusion	At all stages we were open minded to the lay perspective. The role of patient during the semi-structured interviews was not simply to reflect the long list of items generated from systematic reviews. At each stage considerable time was taken by the SAG to reflect on the patient perspective. The time taken in developing the long list minimised ambiguity or queries during later stages of the process	21–23
Reflection/critical perspective	The PPI in this mixed method study was considered and integrated as far as possible into the methods from the very beginning according to best available evidence from the COMET initiative. In the absence of any funding or direct link with major research organisations the COS was developed as per our protocol with consideration given to all elements based on time and resource to maximise patient engagement. A decision to omit patients in the consensus webinars was carefully considered by the SAG which was highlighted in both ethical approval documents and a priori protocol design based upon best available guidance. Since the publication of more recent documents [28] outlining strategies to optimise Patient and public engagement, direct involvement of patients in consensus meetings would be a future consideration	23

### Recruitment of participants

This study captured the views of four stakeholder groups of which the future uptake of the COS is dependent upon. (Patients, Clinicians, Researchers who have conducted previous consent research and Academics working in bioethics). Solicitors and barristers who practice medical negligence law were also included in the process to provide a legal perspective and offer additional potential validity to the core outcome set.

### Semi-structured interviews and Delphi consensus

1. Patients were recruited from a database of patients involved in qualitative research previously conducted by our group investigating the question ‘What is important to patients in the consent process?’ who had indicated that they would participate in future research. These patients had undergone emergency or elective surgery for a wide range of conditions, including day surgery and in-patient surgery for benign and malignant conditions. In addition, an advertisement was posted on the NIHR Peoples in Research website to recruit other willing patient participants.
2. Non-patient groups were approached through the research group’s professional networks, through social media promotion and email contact facilitated by professional bodies such as the Association of Surgeons of Great Britain and Ireland (ASGBI), the Association of Surgeons in Training (ASiT), the Royal College of Surgeons England (RCSEng), the American Society of Bioethics and Humanities and the Department of Legal Services Department of Health and Social Services Northern Ireland among others.

All stakeholder groups were invited to participate via email. A social media page was created by a member of the SAG on twitter© (@IconsStudy) to create direct engagement with professional patient organisations nationally and internationally.

### Generating the survey information

The list of outcomes chosen for prioritisation in this Delphi survey were developed through a series of initial steps. Firstly, a systematic review of outcome reporting in existing trials was conducted to determine which outcomes had been measured previously [7]. Secondly, a systematic review of trial protocols of ‘as yet unpublished’ studies was undertaken to determine if outcome reporting in future trials was likely to be significantly different to that in existing studies. A systematic review of qualitative studies examining patients and clinicians’ attitudes to the consent process determined what mattered most to these stakeholders in the consent process and identified novel outcomes that could be measured. The outcomes identified from these reviews were organised into a consent outcomes taxonomy [27]. Additionally, semi-structured interviews with 41 stakeholders, including patients (n = 12), clinicians (n = 9), consent researchers (n = 10) and medico-legal lawyers (n = 10), explored the opinions on how the quality of the informed consent process should be determined and identified additional outcomes that had not been included in existing research. Semi-structured interviews were conducted by a single member of the SAG (LJC) and a reflective diary was kept. The list of outcomes was reviewed and organised into categories by the study advisory group. Duplicate outcomes were removed and outcomes with different names, but which captured the same phenomenon were amalgamated. A small number of outcomes that were not relevant to the consent process generally were removed from the prioritisation process. The final list of 36 potential outcomes was organised into six categories for prioritisation. These categories (domains) were knowledge, decision making, communication, trust, process, and patient characteristics. The wording of each outcome and explanatory text to describe the meaning of each outcome was developed with the help of the study advisory group’s patient and public representative. To ensure that the items included in the Delphi survey would be understood by all participants and, by patients, four “think out loud” cognitive interviews with lay participants were conducted. These cognitive interviews were undertaken in accordance with recognised methodology in this field [29]. Participants were asked to read aloud the outcome and explanatory text and to describe what they believed the outcome meant. Participants were observed while they read the outcomes to assess for physical cues that might indicate that they did not understand or were unsure such as, grimacing or appearing confused. Where a participant was not clear on the meaning of an outcome, it was discussed with them contemporaneously, and an alternative wording was developed. The wording of the items and their explanatory text was revised following each cognitive interview until no further amendments were deemed necessary. This process was designed to ensure items included in the Delphi would be understood by all participants, particularly patients. The full list of outcomes and accompanying explanatory text is included in Table 2.

**Table 2:** Final list of outcome domains and accompanying explanatory text for Round 1 of Delphi survey

Outcome	Category	Domain name	Help text
1	Knowledge	Measured patient knowledge	Assessment of patient knowledge that they have gained through the consent process. This might involve a written or spoken survey undertaken by another researcher not involved in the consent process
2	Knowledge	Self-rated patient knowledge	For example, asking patients how well informed they feel as a result of this consent process? This might be rated on a scale from 10–1. Where 10 is very well informed and 1 is not informed at all
3	Knowledge	Clinician rated patient knowledge	Clinician (E.g. Doctor) rating of patient knowledge obtained through the consent process. This would be rated by the clinician undertaking the consent process. This might be rated on a scale from 10 – 1. Where 10 is very well informed and 1 is not informed at all
4	Knowledge	Patient rated clinician knowledge	Patient rating of the clinician’s (E.g. Doctor) level of knowledge during the consent process
5	Knowledge	Self-rated clinician knowledge	Clinician (E.g. Doctor) rating of their own knowledge and their ability to answer patient questions during the consent process
6	Knowledge	Patient desire for extra information after the consent discussion	Patient desire for extra information after the consent discussion. For example, searching for info on the internet or speaking with friends and family who have had a similar procedure
7	Knowledge	Patient rated satisfaction with the quality and amount of information disclosed during the consent process	Patient rated satisfaction with the quality and amount of information disclosed during the consent process
8	Decision making	Patient feeling that there was a choice in the consent process	Patient rating of whether they felt they had a choice in the consent process or were aware of alternative options. For example, the choice between surgery and no surgery or a choice between different surgical options
9	Decision making	Confidence in the decision made	Patient rated confidence in their decision to consent or not to consent to the procedure
10	Decision making	Patient rated feeling that the decision to consent or not to the procedure was their own	Patient rated feeling that the decision to consent or not to the procedure was their own
11	Decision making	Patient rating of the influence other people have on their decision to consent. For example, family, friends, other health care workers or other patients	Patient rating of the influence other people have on their decision to consent. For example, family, friends, other health care workers or other patients
12	Communication	Satisfaction with communication	Patient rating of their satisfaction with the quality of communication in the consent process. This communication may be oral, written or audio-visual
13	Communication	External rating of communication	This means another researcher observing the consent process and scoring the quality of communication. This could be by direct observation or by watching a video of the consent discussion
14	Trust	Trust in the clinician	Patient rated level of trust in the clinician guiding them through the consent process
15	Trust	Trust in the hospital	Patient rated trust in the hospital the patient is being treated in
16	Trust	Trust in medicine	Patient rated trust in the science and profession of healthcare
17	Process	Time	This means the total length of time that it takes to complete the informed consent process. This might be measured in terms of minutes or hours
18	Process	Adequacy of time for consent	Patient rated feeling that the length of time for the consent process was neither too rushed nor too long
19	Process	Number of consultations	Number of separate consultations undertaken as part of the consent process
20	Process	Time between consent process and the procedure	How long before the proposed procedure was the consent process conducted
21	Process	Presence of friend or relative	Was a friend, relative or other trusted person present with the patient during the consent process
22	Process	Was the consent process conducted in an emergency situation or in a planned (elective) setting	Was the consent process conducted in an emergency situation or in a planned (elective) setting
23	Process	Consent technique	How the consent process was conducted. For example, did it involve a face-to-face discussion, patient information leaflets, audio-visual aids or other techniques
24	Process	Patient satisfaction with consent process	Patient rated satisfaction with the consent process. This includes the situation for the consent consultation (For example, emergency vs. elective), the timing of the discussion, the number of consultations and the techniques used to undertake the consent process
25	Process	Clinician satisfaction with the consent process	Clinician rated satisfaction with the process used to undertake consent
26	Patient characteristics	Age	Patient age
27	Patient characteristics	Intelligence	For example, IQ or asking patients about their level of education
28	Patient characteristics	Previous experiences of healthcare	Prior experience of surgery and healthcare
29	Patient characteristics	Motivation for surgery	Patient motivation for procedure. Patient preference for a particular procedure before the consent process begins
30	Patient characteristics	Physical state	Assessment of a patient’s physical state which may impact on their ability to consent. For example, level of pain at the time of consent
31	Patient characteristics	Emotional State	Assessment of a patient’s emotional state which may impact on their ability to consent. For example, anxiety level at the time of the consent
32	Patient characteristics	Decision making style	Patient desire to be involved in the decision-making process. For example, happy for others to make decisions on their behalf or want to be in control of all the decisions related to their health care
33	Patient characteristics	Desire for information	Some patients like to have a lot of information. Other patients may not want any information related to their healthcare
34	Patient characteristics	Diagnosis	The medical problem that the patient is being treated for. For example, cancer or benign conditions
35	Patient characteristics	Risk Perception and Risk-Taking Behaviour	The level of risk the patient perceives the procedure to involve. Patient attitude to taking risks in general
36	Patient characteristics	Patient rating of how important they think the consent process is. For example, does the patient feel it is simply a box ticking exercise?	Patient rating of how important they think the consent process is. For example, does the patient feel it is simply a box ticking exercise?

#### The consensus process

An anonymous online Delphi survey was chosen for the consensus process (January-April 2019). The short timeline for completion between rounds aimed to maximise interest and engagement whilst minimising attrition. Summary videos for the Delphi Process (https://www.youtube.com/watch?v=R3bjcEsUS3M) were developed with consultation with the COMET POPPiE Group to provide an audio-visual summary of the study, help participants understand the reason for this research and to optimise recruitment. Participants were asked to rate the importance of each outcome measure in determining the quality of the informed consent process for surgery or another invasive procedure. Outcomes were rated using the Grading of Recommendations, Assessment, Development and Evaluations scale of 1 to 9. In the Delphi exercise, the scale was presented as 1–3 labelled ‘not important’, 4–6 labelled ‘important but not critical’ and 7–9 labelled ‘critical’ [30].

Participants were given the opportunity to suggest additional outcomes not included in the survey at the end of Round 1. These outcomes were reviewed by the study advisory group and duplicate recommendations were removed. Suggested outcomes that were like existing outcomes were excluded and suggested outcomes that were like each other were amalgamated. Participants who suggested an outcome were contacted by email to explain the fate of their suggestion and the reasons for the associated decision, to ensure their ideas had not been misinterpreted and to afford them a right of reply. The included additional outcomes were incorporated into Round 2 of the survey. All outcomes, despite their score, were carried forward to Round 2 to ensure that participants had the opportunity to review their scores for each outcome considering feedback from other participants. Additionally, the number of outcomes identified for prioritisation meant that carrying all outcomes forward to Round 2 would not be unduly onerous. Taking part in Round 1 was a pre-requisite for completion of Round 2. A further video was created to remind participants about the rationale for the survey and explain how Round 2 differed from Round 1. (https://youtu.be/iFoB_Eq0-os) Round 2 provided graphical feedback of the distribution of each stakeholder group’s responses to all participants and reminded them of their previous scores. Presenting participants with feedback from all stakeholders appears to; improve consensus, reduce the variability of responses and improve agreement on those items to keep at the conclusion of the process [31]. Graphical feedback demonstrating the entire distribution of scores was chosen for this study as it was deemed the most easily interpreted form of feedback to demonstrate the spread of scores for each stakeholder group. No specific evidence currently exists to support one form of feedback over another [10].

The distribution of scores for each outcome was calculated as a percentage of total responses. Consensus that an outcome should be considered for inclusion in the COS was defined as 70% or more of total respondents rating it as critical by giving a score in the 7–9 range and no more than 15% rating it is as unimportant by giving it a score of 1–3. Conversely, an outcome would be considered for exclusion if 70% or more of respondents rated it as unimportant and no more than 15% rated it as critical. Additionally, if an outcome met the ‘consensus in’ criteria in three of the four stakeholder groups, but did not reach these criteria overall it was considered for inclusion in the COS. All other measures were thought to be equivocal [11, 32].

#### Patient focus group and consensus webinars

Consensus meetings for patient and other stakeholders were conducted separately. All stakeholders who completed both rounds of the Delphi process were eligible for the consensus webinars and were invited by email. However, many of the patients who had completed the online Delphi survey were not interested in attending such a meeting. For those patients who were prepared to attend a face-to-face meeting, it was not possible to find a mutually convenient date and time to obtain a critical mass. Additionally, the language and non-verbal communication used in such meetings can undermine or exclude patient participants [10]. Indeed, some COS developers recommend that professional and patient consensus meetings should always be conducted separately to allow patients to speak freely and to prevent contamination of their ideas [14]. As such, a patient focus group session was organised with patient participants from the Royal College of Surgeons England (RCSEng) Patient and Lay Group (PLG). The focus group discussion was convened at the Royal College of Surgeons, London on 4 April 2019 over a 1-h period. The PLG was established in 1999 and aims to ensure patient voices are adequately represented in the standards and policies of the RCSEng, raises areas of patient concern to the RCSEng, and advise the RCSEng about the optimal manner to engage patients. This meeting explored patients’ thoughts and perceptions regarding all outcomes prioritised in the Delphi process. Patients were not asked to vote on whether to include outcomes in the final COS. The aim, rather, was to determine patients’ views regarding each of these outcomes meeting consensus in the Delphi process and to use that feedback to inform the discussion during the consensus webinars. Participants were provided with a brief overview of the research and the rationale for the focus group session one week before the session. The patient focus group meeting opened with a brief presentation on the history and development of informed consent for surgery. The results of the online Delphi survey were briefly summarised before discussion was opened to the floor on the outcomes identified for discussion in the consensus meetings. The discussion was semi-structured and participants were asked to indicate their thoughts on all outcomes reaching unanimous ‘consensus in” across all stakeholder groups as well as each of the five borderline outcomes in turn and to highlight any outcomes that they believed were important but not included among the 11 prioritised outcomes.

Two separate consensus webinars were conducted. Participants who completed both rounds of the Delphi survey were invited by e-mail to attend the webinars to produce a consensus panel. Webinars were chosen over an exclusively face-to-face meeting to facilitate participation from a wide geographic area, without the time and financial constraints that international travel would have imposed. These were held on two separate days (one of the webinars was conducted in the morning (UK time) and the other in the afternoon) to facilitate participation of experts from different time-zones and to maximise international attendance and determine the concordance between the panels. An anonymised online voting system was used, and the results were broadcast immediately. In cases where there was no clear consensus result, a discussion was held and a revote was taken. Participants in webinar 2 were not advised how participants in webinar 1 had voted. The decision to include or exclude any outcome was determined by a simple majority across both consensus meetings. Each consensus webinar was recorded using the Adobe Connect software package which recorded the audio, video, online presentation, and online chat generated from the meeting. Voting from each round and salient points of discussion was noted contemporaneously.

## Statistical analysis

DelphiManager Version 3.0 (University of Liverpool) was used to build and manage the Delphi survey. Descriptive statistics and the distribution of scores for each outcome were assessed using SPSS for Windows, Version 24. (IBM Inc., Armonk, NY, USA). Cohen’s kappa scores were calculated to assess the level of agreement between each Delphi round for all outcomes meeting the consensus criteria at the end of Round 2. This was to examine whether consensus might be overestimated because participants with minority opinions do not complete Round 2. Mean and standard deviations were also calculated for these outcomes and an independent *t*-test was performed to detect a difference in the mean scores entered by completers and non-completers between the rounds to evaluate the level of this attrition bias. Graphs for feedback to participants were produced using the R statistical package (R Foundation for Statistical Computing, Vienna, Austria).

## Results

A total of 164 participants completed all elements of Round 1, and a further 5 participants provided usable partial responses: with 125 (76.2%) complete responders in Round 2 and a further 3 participants providing partial responses to that round. Participants completing all elements of the survey came from eight countries and all four key stakeholder groups (Table 3). This included 53 (42.4%) patient participants who completed both rounds. Most respondents from all stakeholder groups originated from the UK.

**Table 3:** Demographics of Delphi participants completing all rounds

Stakeholder						
		Clinicians	Consent researchers/bioethicists	Patients	Solicitors/barristers	Total (%)
Country	Australia	2	1	1	0	4 (3.2)
	Canada	1	0	0	0	1 (0.8)
	Denmark	0	1	0	0	1 (0.8)
	Ireland	4	0	0	0	4 (3.2)
	Netherlands	2	0	0	0	2 (1.6)
	New Zealand	1	0	0	0	1 (0.8)
	UK	41	7	52	6	106 (84.8)
	USA	2	4	0	0	6 (4.8)
	Total (%)	53 (42.4)	13 (10.4)	53 (42.4)	6 (4.8)	125

During Round 1, participants suggested 29 additional outcomes that they believed were not represented in the original survey. These are included in Additional file 1. Review and discussion among the study advisory group resulted in four of these additional outcomes being added to Round 2 (Table 4).

**Table 4:** Additional outcomes suggested by respondents of Round 1 and included in Round 2

Domain	Outcome	Help text
Process	Who is the consenting clinician?	For example; is the doctor seeking consent a consultant (attending) surgeon or a trainee? Is the person undertaking the consent process and the surgical procedure the same?
	Opportunity to ask questions	Did the patient feel there was an opportunity to ask questions during the consent process?
	Shared language of communication	Are the patient and doctor able to communicate in the same language?
Patient characteristics	Patient’s motivation for a particular treatment compared to clinician’s motivation for a particular treatment	Is there a difference between the treatment preference or motivation between the clinician and the patient?

Table 5 displays all outcomes scored in the Delphi process and shows which met the ‘consensus in’ criteria per stakeholder group in both rounds. In most cases, outcomes that met consensus criteria did so in at least two stakeholder groups, but consent technique, diagnosis, shared language of communication, trust in the hospital and trust in medicine were prioritised by patients only. Solicitors alone prioritised measured and self-rated patient knowledge in both rounds of the survey while consent researchers and bioethicists were the only group to prioritise whether the consent process had been conducted in an emergency or elective setting in Round 2, which the lawyer group had rated as critical in Round 1.

**Table 5:**
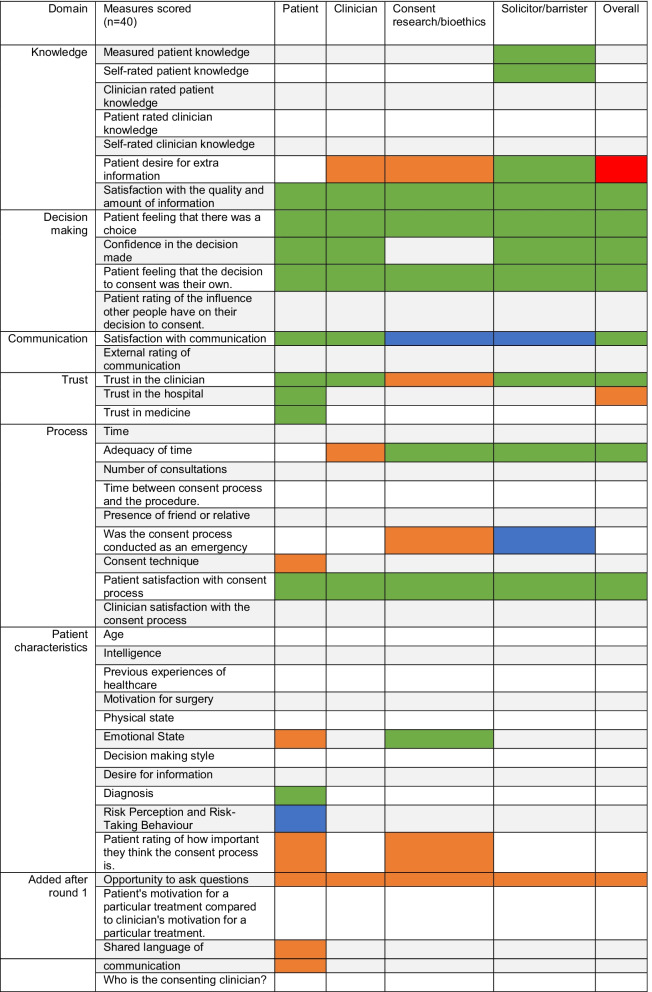
All outcomes scored during the Delphi process

Blue = Met consensus criteria in Round 1. Orange = Met consensus criteria in round 2. Green = met consensus in both Rounds. Red = An outcome not making overall consensus but with consensus in 3 of 4 groups. Consensus defined as > 70% rating ≥ 7 and < 15% rating as ≤ 3

When the responses of participants completing both rounds of the survey were analysed, there were 527 of 4392 opportunities for change instances where a participant moved score categories between the two rounds. Five clinicians and four patients upgraded their rating of an outcome from unimportant to critical between rounds. Conversely, three clinicians and two patients changed their ratings from critical in Round 1 to unimportant in Round 2.

At the end of the Delphi process, 11 of 40 (27.5%) outcomes met the consensus criteria (Table 6). Of these, 6 outcomes met the “consensus in” criteria in each of the four stakeholder groups that took part in the survey, in addition to meeting the criteria overall.

**Table 6:** Outcomes meeting inclusion criteria following two rounds of an online Delphi survey and Cohen’s kappa coefficients to show the degree of agreement between rounds

Domain	Outcome	Participants scoring ≥ 7	Percentage ≥ 7	Participants scoring ≤ 3	Percentage scoring ≤ 3	ƙ R1-R2
Process	Adequacy of time	95	76	2	1.6	0.598
	Opportunity for questions*	112	89.6	0	0	N/A
	Patient satisfaction with process	106	84.8	1	0.8	0.614
Decision making	Patient feeling there was a choice	118	93.7	0	0	0.521
	Patient feeling that the decision was their own	114	91.2	0	0	0.602
	Confidence in the decision made	112	89.6	1	0.8	0.702
Knowledge	Satisfaction with the quality and amount of information	115	91.3	0	0	0.647
	Patient desire for additional information	87	69.0	4	3.2	0.646
Communication	Satisfaction with communication	109	87.2	1	0.8	0.627
Trust	Trust in the clinician	115	92	2	1.6	0.721
	Trust in the hospital	90	72	4	3.2	0.703

^*^Opportunity to ask questions was added to Round 2

These outcomes were:

1. Patient rated satisfaction with the quality and amount of information disclosed during the consent process.
2. Patient believing that there was a choice in the consent process.
3. Patient rated perception that the decision to consent or not to the procedure was their own.
4. Trust in the clinician.
5. Patient satisfaction with the consent process.
6. Opportunity to ask questions.
  There were a further 4 outcomes that while achieving the “consensus in” criteria overall, did not achieve this level of support in all four stakeholder groups. These were:
7. Confidence in the decision made.
8. Satisfaction with communication.
9. Trust in the hospital.
10. Adequacy of time for consent.

Additionally, one outcome, namely, patient desire for extra information after the consent discussion, met “consensus in” criteria in three of the four stakeholder groups but was just short of meeting the criteria when results were analysed overall (69% critical ratings versus 70% required for “consensus in” criteria).

Following discussion among the study advisory group it was determined that the 6 outcomes achieving unanimous consensus should be included in the final COS without the need for prolonged discussion at a consensus meeting. It was also agreed that the other 5 outcomes would be taken forward to the consensus meeting to determine if they would be included in the final COS.

There was substantial agreement in the responses of participants between Round 1 and Round 2 for most outcomes, as indicated by the kappa value > 0.6. The outcomes: “Adequacy of time”, “Patient feeling that there was a choice” and “Patient feeling that the decision to consent was their own” demonstrated moderate agreement between rounds. The proportion of respondents rating these measures as critical increased between rounds, accounting for the lower levels of agreement between rounds but increased agreement between stakeholders.

Comparing mean ratings for outcomes meeting the consensus criteria between participants completing both rounds and those completing Round 1 only does show evidence of attrition bias in the case of “Patient satisfaction with the quality and volume of information” (Table 7). In this case, non-responders rated this outcome significantly lower than responders (7.55 v. 7.98 *p* = 0.03). However, it should be noted that this outcome very clearly made inclusion criteria in both Rounds (Round 1: Critical = 82.8%, Unimportant = 0% and Round 2: Critical = 91.3% Unimportant = 0%) and the difference is small.

**Table 7:** Assessment of attrition bias between completers and non-completers of Round 2

Outcome	Responders round 2			Non-responders round 2			*P*-value
	N	Mean	SD	n	Mean	SD	*t*-test
Adequacy of time	125	7.1	1.4	42	7.31	1.54	0.42
Opportunity to ask questions*	125	8.05	1.13	N/A	N/A	N/A	N/A
Patient satisfaction with process	125	7.52	1.21	42	7.24	1.49	0.22
Patient feeling there was a choice	126	8.27	1.05	41	8.05	1.84	0.34
Patient feeling that the decision was their own	125	8.15	1.11	42	7.95	1.19	0.33
Confidence in the decision made	125	8.09	1.23	42	8.05	1.13	0.82
Satisfaction with the quality and amount of information	126	7.98	1.06	42	7.55	1.31	0.03
Patient desire for additional information	125	6.46	1.47	42	6.93	1.61	0.08
Satisfaction with communication	125	7.95	1.2	42	7.69	1.85	0.29
Trust in the clinician	125	8.21	1.28	42	7.76	1.61	0.07
Trust in the hospital	125	7.34	1.76	42	6.9	2.24	0.19

*Opportunity to ask questions was added to Round 2

## Patient focus group and consensus webinars

The focus group meeting with the RCSEng Patient Liaison (PLG) Group comprised 20 patient representatives from throughout the United Kingdom (8 female and 12 male). Participants endorsed the inclusion of the six outcomes that had reached ‘consensus in’ criteria among all stakeholder groups during the Delphi process in the final COS.. The focus group discussions regarding the remaining 5 borderline outcome were presented to participants in the consensus webinars. The PLG reported that clear verbal communication, avoiding medical jargon and checking for understanding were integral to the consent process. Also, the use of good quality information leaflets would augment the consent discussion. Participants valued the patient rating on the adequacy of time and felt this was a better metric than simply an arbitrary time taken to obtain consent. While members of the group suggested that the need for additional information after the consent discussion and the level of trust patients have in the hospital were important variables, they believed these measures reflected a patient’s personality rather than the quality of the informed consent process. Overall, participants from the RCSEng PLG welcomed a change in the discourse around informed consent and were pleased to see that patient voices were adequately represented in the development of this COS.

The consensus webinars were hosted from the Ulster Hospital Dundonald, Northern Ireland on 6th June 2019, starting at 09:00 BST and 17th June 2019, starting at 14:30 BST. Participants across the two meetings included 12 clinicians, 2 medico-legal lawyers and 3 consent researchers. (Table 8) Most participants originated from the UK with 2 participants from the USA and one each from Australia and Canada.

**Table 8:** Participant characteristics in Consensus Webinars 1 and 2

		Webinar 1	Webinar 2	Total (%)
Stakeholder	Clinician	6	6	12 (70.5)
	Lawyer	1	1	2 (11.8)
	Consent Researcher / Bioethicist	1	2	3 (17.6)
Country	UK	6	7	13 (76.5)
	USA	1	1	2 (11.8)
	Canada		1	1 (5.9)
	Australia	1		1 (5.9)

Participants endorsed the six outcomes that had met unanimous ‘consensus in’ criteria during the Delphi process without further discussion. A summary of the outcome scoring from both webinars is included in Table 9. At the conclusion of both meetings, three of the five outcomes exceeded the 50% threshold to be included in the COS. These were; confidence in the decision made, satisfaction with communication and adequacy of time. This resulted in a final COS consisting of 9 core outcomes (Table 10).

**Table 9:** Outcome voting in both Consensus Webinars

Outcome	Webinar 1 vote in (n = 8)	Webinar 2 vote in (n = 8)	Total vote in (%)	Outcome In/Out
Knowledge: desire for extra information	6	2	8 (50.0)	Out
Decision making: confidence in the decision made	7	3	10 (62.5)	In
Communication: satisfaction with communication	1	8	9 (56.25)	In
Trust: trust in the hospital	0	0	0 (0)	Out
Process: adequacy of time	4	8	12 (75)	In

**Table 10:** Final COS to evaluate interventions designed to improve the informed consent process for surgery

Domain	Outcome
Knowledge	Satisfaction with the quality and amount of information
Decision making	Patient feeling that there was a choice
	Patient feeling that the decision to consent was their own
	Confidence in the decision made
Communication	Satisfaction with communication
Trust	Trust in the clinician
Process	Patient satisfaction with consent process
	Patient rated adequacy of time
	Opportunity to ask questions

## Discussion

This is the first study that has attempted to standardise the important outcomes that should be measured in the informed consent process. It has captured the attitudes of patients, clinicians, lawyers and academics in the field of informed consent internationally. There was a high level of patient involvement throughout the process across various qualitative elements. Patients provided as many complete responses (n = 53 (42.4%) in the Delphi Survey as the clinician group. Collectively, the patients have experience of both emergency and elective surgery for a wide range of conditions, including minor day surgery and major surgery for benign and malignant conditions ensuring a diverse group of patients in terms of age, surgical procedures, and clinical outcomes because of surgery. Furthermore, the consent researcher / bioethicist and clinician groups came from a variety of geographic areas and diverse professional backgrounds and practices.

The prioritisation of outcomes using a Delphi survey and consensus meetings has defined a list of nine outcomes that reflect “what matters” most in the consent process. However, this COS does not preclude the measurement of other outcomes in future consent trials. Despite most of the research to date focusing on knowledge, recall and comprehension as primary outcomes this has not been reflected in the final COS. Measurement of patient knowledge either by objective or subjective means was only rated as critical by the lawyer stakeholder group. Stakeholders preferred a patient satisfaction rating on the quality and volume of information they were provided, as opposed to an attempt to prove whether the information was remembered or understood. The COS aligns with ethical, legal and professional standards and public opinion [33] of seeking to understand what matters to each patient, building trust, communicating effectively, promoting autonomous choice and allowing appropriate time for patients and clinician to make an informed shared decision about treatment. These elements combined highlight that achieving valid consent more likely an reflects a “process” rather than simply satisfying the standards of signing a consent form at a particular point in time.

5 of the outcomes in the final COS have not been reported in any randomised trial of interventions designed to improve the consent process. These are patient rated adequacy of time, opportunity to ask questions, patients feeling like they had a choice in the consent process, that the decision the patient made was their own and trust in the clinician. The fact that these outcome measures, despite being of critical importance to stakeholders in the consent process have not been reported in existing consent research may be because no validated tools exist for measuring some of them, researchers may have considered them but did not think them important to stakeholders, or they have not been considered at all. The remaining outcomes have been measured in existing trials.

This study adds to a paucity of literature on the development of a COS for the evaluation of communication interventions [34]. Additionally this study adds to a limited number of examples where COS developers have used simple statistics to demonstrate stability between rounds and to assess the level of attrition bias. The analysis in this study demonstrated evidence of attrition bias for only one of the eleven outcomes rated as ‘consensus in’. The protocol for the development of this COS was registered prospectively on the COMET database, the full protocol was published prospectively and the parameters for determining consensus were established a priori and mirror the standards used by other COS developers [14, 18, 35]. No deviations from the published protocol were necessary.

Overall, a key question of “what” should be measured in future trials to improve the informed consent process has been satisfied which paves the way to identifying “how” and “when” the outcomes should be measured. This work will follow the COnsensus-based Standards for the selection of health Measurement INstruments (COSMIN) to identify a complementary core measurement set [36, 37].

## Limitations

Despite participation from stakeholders in multiple continents most participants included in this research originate from the UK with a significant proportion of the patient participants coming from a single NHS trust in Northern Ireland. As such, it is possible that these findings may not be generalisable to consent practices in other countries. While several clinicians, bioethicists and consent researchers involved in the studies included live and work in countries outside of the UK, the findings presented may have limited applicability in other settings. As a group of surgeons undertaking this work, it is possible that our professional background may have subjected the design and findings of the study to some unconscious bias. As is the case with all Delphi surveys, limitations of this study could be considered to be responder bias, reduced accountability of views on account of the anonymity afforded to participants and the potential for attrition bias. The outcome “trust in hospital” whilst meeting consensus in both round for patients did not receive any votes at the consensus webinar. This may be reflected by their absence from the consensus webinar component of the study. However, all stages of this research have been subject to clinical, methodological, and patient and public representative oversight. Throughout the qualitative components of this work, great efforts were made to ensure that participants’ voices were being fairly and accurately represented by repeating back our interpretation of what participants had said and agreeing the identification of themes from primary findings among the wider research group. Furthermore, as the study progressed it became clear that the themes identified in a relatively small number of participants during the semi-structured interviews were strongly reinforced by a larger cohort during the Delphi process.

## Implications for clinical practice

Consent to undergo intervention applies to all patients in all specialities of medicine and surgery and therefore has the broadest potential application of any developed core outcome set. The COS from this study has the potential to influence consent practices on a global scale. Many of the outcomes included in the final COS have not been reported in existing consent trials yet these appear to be the aspects that are most important to the key stakeholders in the process. This presents an opportunity to redefine the direction of consent-based research. Regulatory bodies and guideline development groups e.g., National institute of clinical excellence (NICE) endorse the use of core outcome sets and investment to ensure future consent researchers adopt the COS when undertaking and reporting their research is necessary. This would facilitate comparisons between interventions and the synthesis of data while reducing the level of reporting bias. There remain several fields of consent and Shared decision making (SDM) research that may be able to adopt this COS for their own purposes including the trialling of novel communication interventions, Shared decision-making tools and Core Information Sets [8, 38].

## Conclusion

We propose that this COS represents the minimum number of outcomes to report in all future studies of interventions designed to improve the quality of informed consent for invasive procedures. Future work is required to identify the best mechanism of assessment of each core outcome.

## Supplementary Information

**Additional file 1**. Additional outcomes suggested by Delphi participants during Round 1.

## Data Availability

All data generated or analysed during this study are included in this published article.

## Abbreviations

ASGBI: Association of Surgeons of Great Britain and Ireland
ASiT: Association of Surgeons in Training
COMET: Core outcomes in effectiveness trials
COS: Core outcome set
COS-STAD: Core outcome set-standards for development
COS-STAR: Core outcome set-standards for reporting
GMC: General Medical Council
INVOLVE: INVOLVE national advisory group
NHS: National Health Service
NICE: National institute of clinical excellence
PLG: Patient liaison group
PoPPIE: People and patient participation, involvement and engagement
ORECNI: Office of research ethics Northern Ireland
RCSEng: Royal College of Surgeons England
SAG: Study advisory group
SDM: Shared decision making
SEHSCT: South Eastern Health and Social care trust
