# Supplementary material for: Development of a core outcome set for informed consent for therapy: An international key stakeholder consensus study
Source: BMC Med Ethics. 2022 Aug 9;23:79. doi: 10.1186/s12910-022-00820-w (PMC9364552; doi:10.1186/s12910-022-00820-w)
Supplement: Supplementary file 1 — Additional file 1. Additional outcomes suggested by Delphi participants during Round 1. [file 12910_2022_820_MOESM1_ESM.docx]

**Appendix 1: Additional outcomes suggested by Delphi participants during Round 1**

| **Outcome** | **Score** |
| --- | --- |
| Who is the consenting clinician? | 6 |
| Number of clinical encounters during 'consent process' | 8 |
| Time consent discussion held - i.e. OPD vs morning of surgery | 8 |
| Supplemental material (print; electronic) | 5 |
| It is important that the clinician that is obtaining consent has meet the patient before. In my case a clinician obtained consent who I had never met before and never saw again. It was a tick box exercise. | 7 |
| Hospital previous sucess with this procedure? | 9 |
| Statistics on success rates of the proposed procedure / treatment e.g. 70% chance of complete cure | 8 |
| Possible outcomes of not having the proposed procedure / treatment e.g. you will be dead in a month's time | 8 |
| Alternatives to having the proposed procedure / treatment i.e. natural treatments; homeopathy etc. | 8 |
| Costs associated with proposed procedure / treatment if not covered by NHS | 7 |
| Follow up arrangements after the proposed procedure / treatment i.e. every three month check up for a year etc | 7 |
| Recovery time to recover if having surgery | 8 |
| ***Not an outcome suggestion; but a comment: please do not equate intelligence with education. Education level would be the better title for that measure.*** | 8 |
| Patient's motivation for a particular treatment compared to clinician's motivation for a particular treatment. | 7 |
| Success rate of the operation | 8 |
| After care; I called with Chemist shop for advice on condition of wound and treatment | 7 |
| Patient understanding if what they are NOT consenting to ; when they consent for surgery | 9 |
| Clinician understanding of what patient is NOT consenting to; when they consent for surgery | 9 |
| Satisfaction of both patient and clinician | 8 |
| Understanding of what has been said ie speaking back about their understanding of the message; this will ascertain comprehension | 7 |
| Trust in health staff; necessity of surgery | 7 |
| Patient understanding the options available and relative risks / benefits | 7 |
| If surgeon cannot offer alternative surgical approach which better for patient; that option being available elsewhere | 6 |
| Patient ability to return to decision making process with clinician if more information sought | 7 |
| Opportunity to ask questions | 8 |
| Patient overall feeling that effective consultation helped him/her through the surgical process | 8 |
| Shared language of communication | 6 |
| Time between discussion and decision | 8 |
| Provision of written support materials for the patient | 9 |
